# Permalloy-Based Thin Film Structures: Magnetic Properties and the Giant Magnetoimpedance Effect in the Temperature Range Important for Biomedical Applications

**DOI:** 10.3390/s17081900

**Published:** 2017-08-17

**Authors:** Anna A. Chlenova, Alexey A. Moiseev, Mikhail S. Derevyanko, Aleksandr V. Semirov, Vladimir N. Lepalovsky, Galina V. Kurlyandskaya

**Affiliations:** 1 Laboratory of magnetic sensors, Ural Federal University, Ekaterinburg 620002, Russia; anniaally@gmail.com (A.A.C.); vladimir.lepalovsky@urfu.ru (V.N.L.); 2 Pedagogical Institute, Irkutsk State University, Irkutsk 664003, Russia; moiseev.al.an@gmail.com (A.A.M.); mr.derevyanko@gmail.com (M.S.D.); semirov@mail.ru (A.V.S.); 3 Departamento de Electricidad y Electrónica and BCMaterials, Universidad del País Vasco UPV/EHU, Bilbao 48080, Spain

**Keywords:** magneto-impedance, magneto-elasticity, magneto-electricity, magnetic sensors

## Abstract

Permalloy-based thin film structures are excellent materials for sensor applications. Temperature dependencies of the magnetic properties and giant magneto-impedance (GMI) were studied for Fe_19_Ni_81_-based multilayered structures obtained by the ion-plasma sputtering technique. Selected temperature interval of 25 °C to 50 °C corresponds to the temperature range of functionality of many devices, including magnetic biosensors. A (Cu/FeNi)_5_/Cu/(Cu/FeNi)_5_ multilayered structure with well-defined traverse magnetic anisotropy showed an increase in the GMI ratio for the total impedance and its real part with temperature increased. The maximum of the GMI of the total impedance ratio ΔZ/Z = 56% was observed at a frequency of 80 MHz, with a sensitivity of 18%/Oe, and the maximum GMI of the real part ΔR/R = 170% at a frequency of 10 MHz, with a sensitivity of 46%/Oe. As the magnetization and direct current electrical resistance vary very little with the temperature, the most probable mechanism of the unexpected increase of the GMI sensitivity is the stress relaxation mechanism associated with magnetoelastic anisotropy.

## 1. Introduction

Magnetic sensors were of great interest in electrical engineering and biomedical research during the past four decades [1,2,3,4]. The existence of many types of magnetic field sensors is a consequence of very broad technological demands. For each particular application a combination of particular parameters is required, among which one can mention sensitivity with respect to the applied field, full-scale range, linearity, hysteresis, temperature coefficient of sensitivity, bias stability, offset features, noise, resistance to environmental factors, power consumption, size, cost, long-term stability (including stability to different temperature conditions), etc., [5,6,7,8].

For magnetic sensors based on thin film multilayered structures, which are promising nanomaterials for weak magnetic field sensors, and especially biosensors, the long-term stability is an underdeveloped area with very few studies. Here, it is necessary to distinguish thermal treatments at elevated temperatures for relaxation and modification of the effective magnetic anisotropy and sensitive element response at different temperatures. In the first case all measurements are done at room temperature but, in the second, they are measured for different elevated temperatures [9,10]. Selected non-systematic data on the temperature dependence of giant magneto-impedance (GMI) of wires, amorphous, and nanocrystalline ribbons can be also found in the literature and, very recently, this point become a hot spot of interest [10,11].

GMI-based sensitive elements caused special interest of multidisciplinary research groups due to their high sensitivity with respect to the external magnetic fields [12,13,14]. The magnetoimpedance phenomenon corresponds to the change in complex impedance (Z) of a ferromagnetic conductor submitted to an external magnetic field (H) [15,16]. A high GMI effect is observed in samples with well-defined effective magnetic anisotropy and high dynamic magnetic permeability [15]. Thermal treatments are the effective way to improve the effective magnetic anisotropy via either formation of induced magnetic anisotropy or stress relaxation [12,17]. Temperature fluctuations are always present in the environment and they determine the operating conditions of magnetic field detectors.

The best GMI configuration in the case of a thin film element is a multilayered ferromagnet/conductor/ferromagnet structure with well-defined transverse magnetic anisotropy [14,18]. The thicknesses of the ferromagnetic layers and the central conductive lead for operating frequencies of the order of several tens of MHz should be at least 0.5 μm [14,18]. However, it is not possible to obtain magnetically-soft permalloy films of this thickness because of the existence of the transition to a “transcritical” state [19,20,21]. A technological solution of this problem, the nanostructuring of ferromagnetic layers, was proposed and successfully demonstrated by different groups [21,22,23].

At the present moment studies of the temperature dependence of GMI multilayered nanostructures are not available in the literature. The main reason are technical difficulties of the measurement of the high-frequency impedance at elevated temperatures. At the same time, it is necessary to test magnetically-sensitive elements for biomedicine in the temperature interval up to 50 °C corresponding to the living system conditions and heating therapies [24,25]. Although traditional multilayered structures are asymmetric by their nature, as the materials are deposited onto solids (glass, silicon, etc., substrate), in addition, new GMI materials with asymmetry of the ferromagnetic layers “above” and “below” the central current lead are proposed for sensor applications [26,27]. All features of topological asymmetry are certainly contributing to the parameters of anisotropic thermal conductivity of the whole system and its long-term stability.

**Figure 1:**
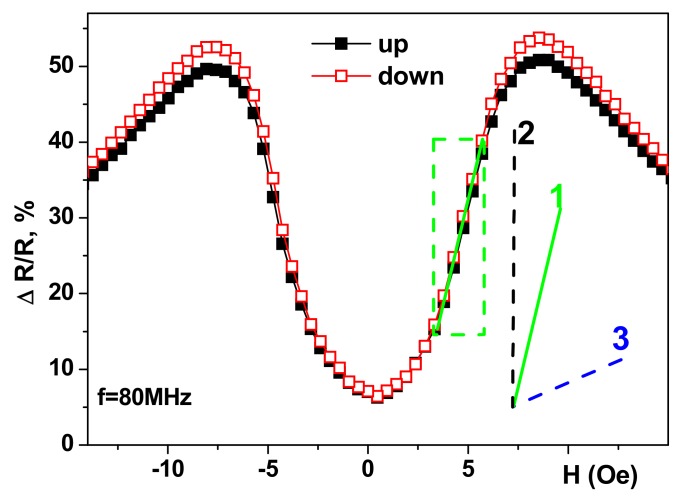
Field dependencies of the real part of the total impedance ratio for the frequency of 80 MHz (25 °C measurements): glass/[Cu(3 nm)/FeNi (100 nm)]_5_/Cu(500 nm)/[Cu(3 nm)/FeNi(100 nm)]_5_. A section of the linear dependence of a ΔR/R (working interval) is shown by the light green dashed line. Line 1 is the slope for the linear part of the maximum sensitivity for glass/[Cu(3 nm)/FeNi(100nm)]_5_/Cu(500 nm)/[Cu(3 nm)/FeNi(100 nm)]_5_ multilayer; line 2 is given for comparison, it corresponds to 350%/Oe sensitivity; line 3 is given for comparison, it corresponds to 2%/Oe sensitivity. “Up” is the GMI branch in an increasing field and “down” in a decreasing magnetic field.

Each type of magnetic sensor has a number of specific requirements. One of the most important parameters is the sensitivity with respect to an externally-applied field. Figure 1 shows a typical magnetoimpedance response with respect to an applied field (for the real part of the total impedance, R). ΔR/R = 100% × [R(H) − R(H_max_)]/R(H_max_), where H_max_ = 150 Oe.

A section of the linear dependence of a ΔR/R with a maximum slope is called the working interval. Line 1 corresponds to a glass/[Cu(3 nm)/FeNi (100 nm)]_5_/Cu(500 nm)/[Cu(3 nm)/FeNi(100 nm)]_5_ multilayer, which we propose to study in the present work; line 2 corresponds to the glass/[Ti(6 nm)/FeNi(100 nm)]_4_/Cu(400 nm)/[Ti(6 nm)/FeNi(100 nm)]_4_ multilayer with one of the best values of GMI sensitivity reported in the literature [28]; line 2 is the slope for the maximum sensitivity for giant magnetoresistance detectors [8,13,29]. One can see that GMI sensors offer extraordinary sensitivity with respect to an applied magnetic field. Although the FeNi/Ti-based structure shows the highest GMI sensitivity at room temperature it requires more complex preparation technology as three different materials are involved. Copper is a well-known material for very good thermal conductivity which might be very important in the case of working temperatures slightly above room temperature. As the studies of the temperature dependencies of GMI for FeNi-based multilayers are very limited in the literature the further development of specialized sensors are conditioned by the development of this part of the research. In the present work, the temperature dependencies of the magnetic parameters and giant magnetoimpedance were studied for the case of Cu/FeNi-based multilayered structures in the temperature range important for technological and biomedical applications.

## 2. Experimental

Multilayered thin film structures based on Fe_19_Ni_81_ permalloy with close to zero magnetostriction were prepared by the ion-plasma sputtering technique onto Corning glass or polymer substrates at room temperature. The background pressure was 3 × 10^−7^ mbar and the argon pressure during deposition was 3.8 × 10^−3^ mbar. The deposition rate for FeNi layers was 28 nm/min for FeNi and 25 nm/min for Cu layers. The deposition rates and the thicknesses of the films were determined in a calibration procedure with sharp step 100 nm samples and atomic force microscopy. Flexible polymer substrates were cycloolefine Zeonor ZF14 100 μm thick commercial film (ZEON EUROPE GMBH, Düsseldorf, Germany) protected by an additional covering, which was removed in a clean room just prior to deposition. Structures S and A were deposited onto a glass substrate (G). Structure AS was deposited onto a flexible cycloolefine polymer substrate widely used for microfluidic device fabrication [30].

**Figure 2:**
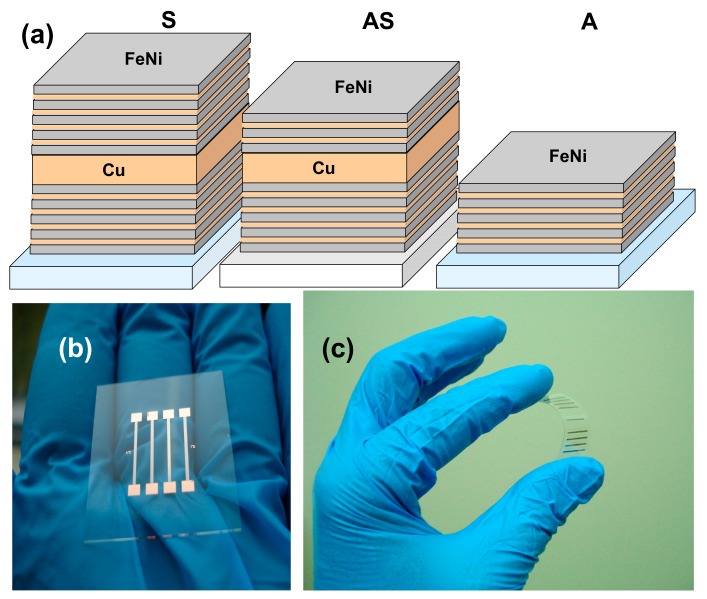
Schematic description of the GMI multilayered structures (**a**). General view of GMI-sensitive elements deposited onto glass (**b**) and flexible polymer (**c**) substrates.

For GMI-sensitive element formation metallic masks were used during the multilayered structure deposition in order to obtain elongated stripes with a geometry of 11.0 mm × 0.5 mm. The following samples were prepared (Figure 2): S − G/[Cu(3 nm)/FeNi (100 nm)]_5_/Cu(500 nm)/[Cu(3 nm)/FeNi(100 nm)]_5_; AS − CO/[Cu(3 nm)/FeNi(100 nm)]_5_/Cu(500 nm)/[Cu(3 nm)/FeNi(100 nm)]_3_; A − G/FeNi(100 nm)/Cu(3 nm)]. Four or eight sensitive elements were deposited at a time. Figure 1 shows a general view of the series of GMI elements deposited onto both types of substrates.

A magnetic field of about 100 Oe was applied during the GMI stripe deposition along the short side of the rectangular GMI element aiming to create a well-defined uniaxial magnetic anisotropy.

Magnetic hysteresis loops of the multilayered structures were measured for 5.0 mm × 0.5 mm samples using a SQUID magnetometer for the same temperatures of 25 °C to 50 °C as those used in the GMI studies. All measurements were done along the hard magnetization direction corresponding to the long side direction of the GMI element. Such a temperature interval was selected being the interval of biological applications importance [24]. In selected cases the Akulov-Bitter technique was used in order to visualize the surface magnetic domains [31]. The measurements of the direct current electrical resistance (R_dc_) was carried out according to the standard procedure of the four points technique for a current intensity of 10 mA.

The GMI measurements were performed using a radio-frequency technique [10,13]. The samples were installed into a 50 Ω “microstrip” line using silver paint. The total impedance (Z), real (R), and imaginary (X) parts of the total impedance (Z = R + *i*X) were obtained from the measured S_11_ parameters after calibration and mathematical subtraction of the test fixture contributions. Z, R, and X were automatically measured in the frequency range (f) of an alternating current of 0.1 MHz to 110 MHz under an external axial magnetic field using Agilent 4294A impedance analyzer. An external quasistatic magnetic field was produced by a pair of Helmholtz-calibrated coils with compensation for geomagnetic and technogenic magnetic fields. The sample holder consisted of a micalex base with mechanically-fixed measuring contacts made of brass with silver plating connected to the analyzer. Heating of the sample was done by the air flow with a fuser, including an air blower, heating element, thermally-insulated duct, and two thermoelectric transducers for measurements and control of the temperature.

The GMI ratio for total impedance and its real part in an external magnetic field was calculated as follows: ΔΖ/Ζ = 100% × [Z(H) − Z(H_max_)]/Z(H_max_), ΔR/R = 100% × [R(H) − R(H_max_)]/R(H_max_), where H_max_ = 150 Oe. The maximum value for each frequency was denoted as ΔΖ/Ζ_max_ for the total impedance and ΔR/R_max_ for its real part. An important characteristic of the GMI is its maximum sensitivity with respect to the external magnetic field. The sensitivity of the GMI was: S(ΔΖ/Ζ) = ΔΖ/Ζ/ΔH or S(ΔR/R) = ΔR/R/ΔH, where ΔH = 0.1 Oe. For the stable operation of the sensor element, it is necessary to have a section of linear dependence ΔZ/Z(H) on the order of 1–3 Oe, called the working interval, its center denominated as the operating point [31].

## 3. Results and Discussion

**Figure 3:**
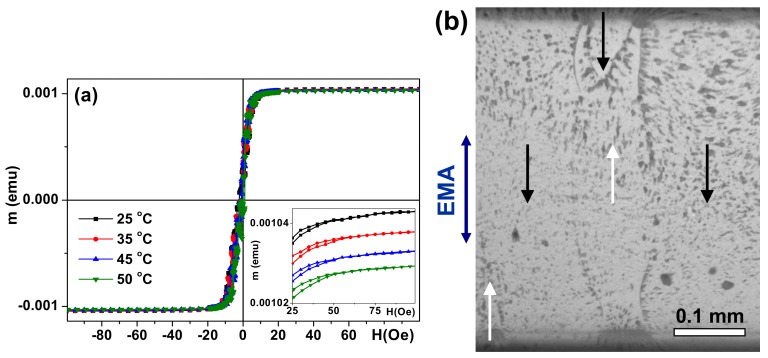
S-type multilayered structure. SQUID hysteresis loops measured along the hard magnetization axis in the plane of the film for selected temperatures: the inset shows field dependencies of magnetic moments (m) for selected temperatures at better resolution (**a**). The surface magnetic domain structure revealed by Akulov-Bitter technique with ferrofluid at room temperature (25 °C) in the S sample: black and white arrows indicate the orientation of the magnetization in the domains, and the easy magnetization axis (EMA) is oriented in the same direction as the direction of the applied field during thin film deposition, being parallel to the short side of the elongated stripe (**b**).

Figure 3a shows the hysteresis loop of the S sample measured in the hard magnetization direction as an example. All multilayeres (S, AS, A) were very soft ferromagnets with a coercivity value less than 1 Oe. They were also characterized by uniaxial transverse magnetic anisotropy with an easy magnetization axis coinciding with the direction of application of a weak external magnetic field during sample deposition. The surface magnetic domain structure also confirms the uniaxial transverse magnetic anisotropy formation (Figure 3b). As expected for permalloy, the temperature dependence of the magnetization (m) in the 25 °C to 50 °C interval was weak. An increase in temperature caused a very small decrease of the magnetic moment in the saturation field (Figure 3a, inset). The shapes of the hysteresis loops were almost the same, with very low coercivity on the order of 1 Oe.

In all cases under consideration the magnetic anisotropy field was close to 7 Oe. Several factors contribute to the magnetic behavior of the samples and must be considered. On the one hand, the magnetic properties of each individual layer must be taken into account. The type of domain walls depends on the thickness of the permalloy films [32]. A single-layered 100 nm thick FeNi film displays two types of magnetic domain walls: 180 Néel walls with cross ties and stray-field free asymmetric mobile-vortex Bloch walls [32,33]. The different types of domain walls may influence the interaction between layers [25,32]. Although comparative analysis of the hysteresis loops and surface magnetic domains (Figure 3) indicates the dominant contribution of transverse uniaxial effective anisotropy, the existence of closure domains and a supposedly small deviation of the magnetization vector out of plane of the multilayered structure are also observed.

**Figure 4:**
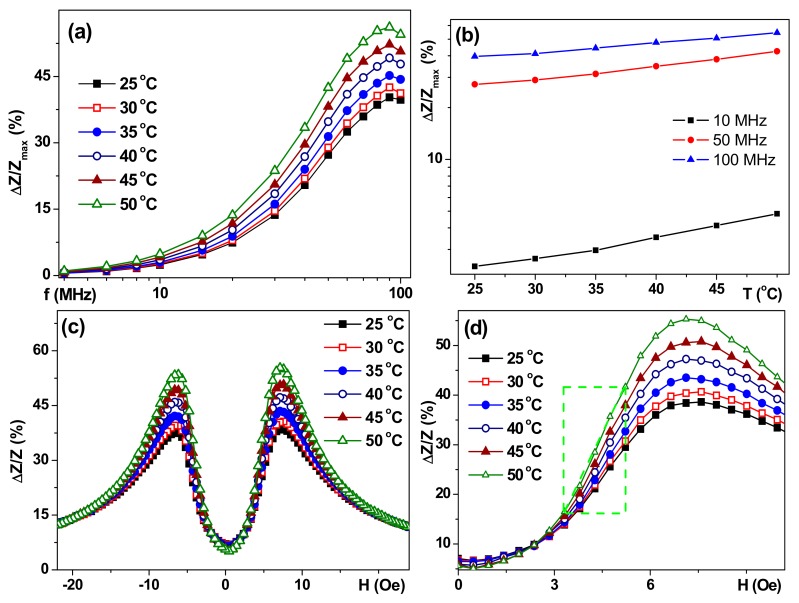
S-type multilayered structure. Frequency dependencies of the ΔZ/Z_max_ GMI ratios for selected temperatures (**a**). Temperature dependencies of the ΔZ/Z_max_ GMI ratio for selected frequencies (**b**). Field dependencies of the GMI ratio at f = 80 MHz (**c**,**d**) at different temperatures. A section of linear dependence of a ΔZ/Z (working interval) is shown by the light green dashed line (**d**). Only one branch of the GMI curve after saturation in the high positive field is shown for (**c**,**d**) cases.

Figure 4a,b show the frequency dependencies of the maximum value of the GMI ratio of the total impedance for selected temperatures in the temperature range important for technological and biomedical applications. ΔΖ/Ζ_max_(f) is typical for a symmetric structural shape [18,30] in the particular cases under consideration, i.e., with plateaus for maximum values in the frequency range of 70 MHz to 100 MHz.

High-frequency electrical impedance of the soft ferromagnet depends on the changes of the transverse magnetic permeability due to the skin effect, which describes the non-uniform penetration of the electromagnetic field associated with an alternating current flowing through the material. The exponential decrease of the amplitude of the fields from the surface of the sample can be described by the skin penetration depth (δ): δ = (πfσμ)^−1/2^, where f is a frequency of the driving current, σ (the conductivity of the material) and μ (the transverse magnetic permeability) [34]. The skin effect contributes to the change of the impedance of the sample because it changes the effective cross-section available for the alternating current to flow either by frequency change or/and the change of the magnetic permeability. In the case of temperature variations all (f, σ, and μ) parameters can contribute to the skin penetration depth features and, therefore, the impedance variations.

Despite the fact that the shape of ΔΖ/Ζ_max_(f) varies insignificantly for the temperature range under consideration, the temperature increase leads to a significant increase in ΔΖ/Ζ_max_ for each fixed frequency: Figure 4 shows a linear increase of Log(ΔΖ/Ζ_max_(T)) for low, intermediate, and high frequencies. This observation is quite unexpected in light of a very slight decrease in the magnetic moment changes (Figure 2a). The frequency dependence of ΔR/R_max_(f) (not shown here) was also typical for a symmetric structure. The shape of the frequency dependence ΔR/R_max_(f) changed very little, but for each fixed frequency an increase in temperature resulted in a significant increase in ΔR/R_max_.

**Figure 5:**
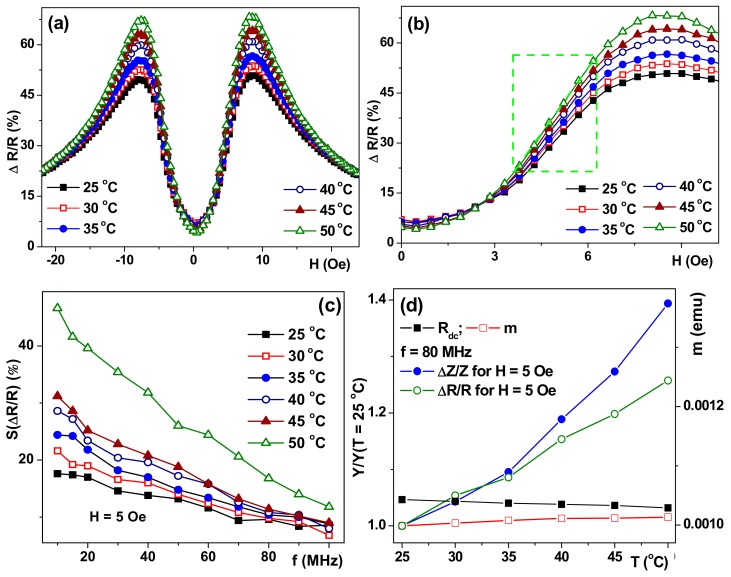
All data for sample S. Field dependencies of the GMI ratio for thereal part of the total impedance for at f = 80 MHz measured at different temperatures (**a**). A section of linear dependence of a ΔR/R (working interval) is shown by the light green dashed line (**b**). Frequency dependencies of maximum sensitivity with respect to an applied field in H = 5 Oe for the ΔR/R ratio (**c**). Temperature dependencies of Y/Y(T = 25 °C) parameters, where Y = R_dc_, m, ΔZ/Z, and ΔR/R (**d**).

Figure 5 shows field dependencies of the ΔR/R ratio (only one branch of the GMI curve after saturation in the high positive field is shown for simplicity) for a frequency of 80 MHz for which both ΔΖ/Ζ and ΔR/R are close to the absolute maximum. One can clearly see the both GMI maxima (in positive and negative magnetic fields) have very similar shapes and values. The maximum difference of the GMI ratios measured at different temperatures appears in the external field close to the anisotropy field (Figure 5a). At f = 80 MHz, the maximum ratio of the total impedance ΔZ/Z_max_ with increasing temperature by 25 °C increases by 18%, and ΔR/Rmax increases by 19%. In fact, the thermal sensitivity of ΔZ/Z and ΔR/R GMI ratios near the anisotropy field, or below it (work point about 5 Oe for work interval of 3.3 Oe to 5.7 Oe), can be used for the development of a temperature sensor. Important remark: gentle heating in the temperature range under consideration did not cause any heat treatment, i.e., after heating up to 50 °C the GMI parameters returned back to their values previously observed at T = 25 °C in the frame of the accuracy of the experimental technique (about 1.5%).

As it was mentioned before, the sensitivity with respect to an external field is the most important senor technological parameter. The sensitivities of the GMI of S structure for the total impedance S(ΔZ/Z) and its real part S(ΔR/R) was calculated in a field of 5 Oe (Figure 4 and Figure 5). The obtained dependencies shows an increase in S(ΔZ/Z) and a decrease in S(ΔR/R) with a frequency increase for all temperatures under consideration. An increase in the sensitivity of both GMI ratios with the increase of the temperature was also observed for each fixed frequency. It should be noted that the sensitivity of the GMI ratio of the total impedance at all investigated frequencies did not exceed 10%/Oe at room temperature (25 °C) and 20%/Oe at 50 °C, the greatest increase in sensitivity was observed in the frequency range of 70 MHz to 80 MHz. The sensitivity of the GMI real part ratio (Figure 5b) shows a monotonous decrease with an increase of the frequency with the greatest difference in the low-frequency region on the order of 10 MHz. An increase of the temperature leads to a significant increase in sensitivity: S(ΔR/R) at a temperature of 50 °C was 46%/Oe at a frequency of 10 MHz (Figure 5c).

In order to understand the overall change of GMI responses with the temperature increase we have measured not only the temperature dependence of the magnetic moment, but also R_dc_(T) resistance changes. Figure 5d shows such a comparison. As it was dicussed above, m(T) dependence cannot be decisive in understanding the GMI behavior. The observed R_dc_ increase with the temperature grows for all structures was of the order of 2% with respect to the room temperature value, whereas ΔZ/Z increased by 10% for structure A, 15% for AS structure and 40% for structure S. Comparative analysis of GMI, R_dc_, and m(T) temperature dependencies lead us to the conclusion that the observed increase of GMI ratios and their sensitivities can be understood as the relaxation of stresses associated with magnetoelastic anisotropy. The mechanism of partial relaxation of internal stresses seems to be connected with the difference in the thermal extension coefficients of the film and substrate.

As it was mentioned before, the sensitivity with respect to the applied field is very important, but not the only requirement for magnetic sensor applications. As the temperature studies for glass substrate-deposited multilayers are very limited, and they are absent for the case of flexible substrates, we deliberately analyzed some very different examples in order to take the first step in this direction, establishing both similarities and differences.

**Figure 6:**
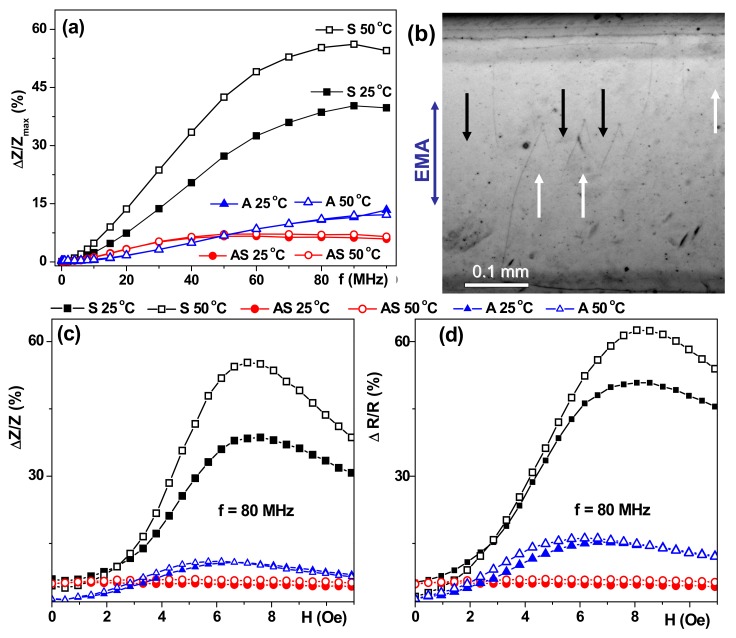
Frequency dependence of the maximum value of the ΔZ/Z ratio for low and high temperatures for S − G/[Cu(3 nm)/FeNi (100 nm)]_5_/Cu(500 nm)/[Cu(3 nm)/FeNi(100 nm)]_5_, AS − CO/[Cu(3 nm)/FeNi(100 nm)]_5_/Cu (500 nm)/[Cu(3 nm)/FeNi(100 nm)]_3_, and A − G/FeNi(100 nm)]5/Cu(3 nm) multilayered structures (**a**). Magnetic domains revealed by the Akulov-Bitter technique with a ferrofluid at room temperature (25 °C) in the AS sample: black and white arrows indicate the orientation of the magnetization in the domains, the easy magnetization axis (EMA) is oriented in the same direction as the direction of the applied field during thin film deposition, being parallel to the short side of the elongated stripe (**b**). The field dependence of ΔZ/Z (**c**) and ΔR/R ratios (**d**) for two selected temperatures.

Classic symmetric GMI structure S showed the best GMI parameters in comparison with asymmetric AS or A structures (Figure 6). Asymmetric structures showed a much smaller GMI value and its sensitivity with respect to the external magnetic field for all temperatures. Such a decay of the magnetoimpedance characteristics is connected with three different phenomena. The first one can be understood taking into account the electrodynamic origin of the GMI and the connection with the skin-effect: a high degree of asymmetry causes additional non-uniformity of the current distribution and a different contribution of the magnetostatic interaction between the layers [23,35]. The second one is related to stress accumulation and anisotropy distribution characteristics for the multilayers deposited onto flexible substrates [36,37,38]. The last one is related to the non-uniform thermal conductivity of the heterogeneous structure, which is especially problematic for polymer substrates. At the same time, polymer substrates offer the opportunity of at least halving the weight of the device in comparison with silicon-based sensitive elements, and despite the difficulties with texturing of thin films deposited onto flexible substrates and control of the uniaxial magnetic anisotropy, they are a very promising option for present day electronics [37,38].

Although ΔZ/Z_max_(f) dependence with a maximum typical for multilayers was observed for an AS structure with a lower degree of asymmetry the impedance variation was too small to be an effect of wide practical importance. Even so, weak temperature dependence in the temperature range of 25 °C to 50 °C combined with about 5%/Oe sensitivity can be useful for particular applications in microfluidic biotechnological devices. The surface magnetic domains were quite different compared with the domain structure in the S sample case: the most significant difference is the appearance of “head-to-head” domain configurations (Figure 5b). At the same time one can still clearly see that EMA orientation corresponds to the orientation of the external field applied during sample deposition. This more complex type of magnetic domain structure of the top layer is consistent with the supposition about stress accumulation in the multilayers deposited onto flexible substrates.

The ΔZ/Z_max_(f) behavior observed for the A structure can be interpreted as the displacement of the ΔZ/Z_max_ maximum value in the region of higher frequencies with respect to the maximum position observed for the S multilayer. ΔZ/Z_max_(f) dependencies for non-symmetric structures are similar to each other with very weak temperature dependence. Similar tendencies were observed in the ΔR/R_max_(f) case and the highest sensitivity was observed for the symmetric S GMI multilayer. The frequency of 80 MHz was chosen for the analysis of the field dependencies of the total impedance in all structures, since it showed the GMI maximum for the total impedance ratio for the S structure (Figure 6). ΔZ/Z and ΔR/R field dependencies for a symmetric structure are characterized by the presence of two pronounced maxima in a positive and negative field close to the anisotropy field known from SQUID magnetometry. The peaks on the ΔZ/Z(H) curves for different temperatures roughly coincide in their position. Peaks on the ΔR/R (H) curves for a high temperature are slightly shifted to the region of large fields.

There are just a few theoretical studies on the GMI in asymmetric structures [39] and further accumulation of the experimental results is necessary for better understanding of the advantages and disadvantages of flat asymmetric structures deposited onto rigid and flexible substrates. The last case is especially important for magnetic biodetection, in which either superposition of the stray field of magnetic markers or changes in the magnetic permeability of top covering layer can be detected by a magnetic field sensor [1,26,40]. The next step is to incorporate the field sensor into microfluidic system directly.

Although the obtained results are very promising for the design of the magnetic biosensors, at this point we were not able to design GMI prototypes for ferrofluid, flowing magnetic markers, magnetic label, or ferrogel detection [40,41,42,43,44] at elevated temperatures. Design and testing of such prototypes require sets of different additional studies of the temperature dependence of the magnetic properties of magnetic nanoparticles, magnetic markers, ferrofluids, and ferrogels under the conditions of constant weight and nanoparticle concentrations [43]. As the magnetic properties of both GMI-sensitive elements and biological samples (or samples mimicking the biological properties) with magnetic nanoparticles may depend on the temperature, one must search for synergetic combinations of the properties of two different materials in order to ensure the best performance.

As an example of possible applications of a GMI-based magnetic field sensor designed onto a flexible substrate we can share the idea of the monitoring of the magnetic response of injected nanoparticles for hyperthermia application. Sensitive elements can be located onto the skin area in close proximity to a tumor. As soon as the accumulation is detected, personnel may consider starting with the hyperthermia therapy. In this particular case the functionality at about 36 °C, a flexible substrate, and sensitivity to the magnetic permeability of the tissue are necessary.

## 4. Conclusions/Outlook

Multilayered Fe_19_Ni_81_/Cu-based symmetric and asymmetric magnetoimpedance structures with transverse-induced magnetic anisotropy were prepared by the conventional sputtering technique and studied in the temperature interval of 25 °C to 50 °C corresponding to the interval of many technological applications, including magnetic biosensing.

An increase in the GMI ratio of the total impedance and its real part for glass/[Cu(3 nm)/FeNi(100 nm)]_5_/Cu(500 nm)/[Cu(3 nm)/FeNi(100 nm)]_5_ structure was observed for increasing temperature. The maximum of ΔZ/Z = 56% was measured at f = 80 MHz, with a sensitivity of 18%/Oe. The maximum of the GMI ratio of the real part was ΔR/R = 170% at f = 10 MHz, with 46%/Oe sensitivity.

As the magnetic permeability and electrical resistance varied very little with temperature, they could not be responsible for the GMI increase. The mechanism of partial relaxation of internal stresses due to the difference in the thermal extension coefficients of the film and substrate was discussed. An increase in the dynamic magnetic permeability of multilayers was proposed as the most probable reason for the increase of the sensitivity of the GMI effect in Fe_19_Ni_81_/Cu-based structures.

Although more studies are necessary for the development of GMI multilayered-structure biosensor prototypes operating at elevated temperatures, the obtained results indicate the possibility of the development of such prototypes in the nearest future.
